# Micro-computed tomography reconstructions of tibiae of stem cell transplanted osteogenesis imperfecta mice

**DOI:** 10.1038/sdata.2018.100

**Published:** 2018-05-29

**Authors:** Anna M. Ranzoni, Michelangelo Corcelli, Timothy R. Arnett, Pascale V. Guillot

**Affiliations:** 1 Institute for Women's Health, University College London, WC1E 6AU London, UK; 2 Department of Cell & Developmental Biology, University College London, WC1E 6BT London, UK

**Keywords:** Mesenchymal stem cells, X-ray tomography, Regeneration

## Abstract

Micro-computed tomography (micro-CT) is commonly used to assess bone quality and to evaluate the outcome of experimental therapies in animal models of bone diseases. Generating large datasets is however challenging and data are rarely made publicly available through shared repositories. Here we describe a dataset of micro-CT reconstructed scans of the proximal part of 21 tibiae from wild-type mice, osteogenesis imperfecta mice (homozygous oim/oim) and oim/oim mice transplanted with human amniotic fluid stem cells. The dataset contains, for each sample, 991 8-bit Bitmap reconstructed images and a 3D reconstruction of the bone in the PLY format, available at the online repository Figshare. In line with the increasing effort to make scientific datasets open-access, our data can be downloaded and used by other researchers to compare their observations with ours and to directly test scientific questions on osteogenesis imperfecta bones without the need to generate complete datasets.

## Background & Summary

The availability of rodent models of bone diseases is essential for the fast progression of biomedical research and discovery of novel therapeutics. These models are used to mimic a variety of bone conditions, including osteoarthritis, osteoporosis, age-related bone loss and osteogenesis imperfecta^1–3^. Osteogenesis imperfecta (OI) is a rare genetic disease with prenatal onset caused by mutations in the genes involved in collagen biosynthesis^4,5^. The most widely used model of OI is the *oim* model (OI mouse, B6C3Fe a/a-Col1a2^oim^/Col1a2^oim^), which carries a spontaneous mutation in the collagen type 1 alpha 2 chain (Col1α2) gene, leading to the absence of this protein and the consequent production of a defective homotrimeric α1[I]_3_ collagen molecule instead of the normal heterotrimeric α1[I]_2_α2[I]_1_ one^6–8^. The *oim* model exhibits characteristics of the severe human form such as reduced size, skeletal fragility, frequent fractures, and abnormal bone microarchitecture, including reduced cortical and trabecular thickness, reduced trabecular number and increased porosity. Microstructural analyses of bones are essential to assess these morphological parameters and micro-computed tomography (micro-CT) represents an invaluable tool, allowing 3-dimensional analysis of bones.

Since its introduction in the 1980s (ref. 9), micro-CT has become an indispensable tool across the skeletal research community for morphological analyses of both cortical and trabecular bone, leading to the wide-spread commercialisation of scanners. Micro-CT is a reliable and reproducible technique^10–12^ which has many advantages over 2D histomorphometry. For example, it allows 3D quantitative analysis of a much larger portion of the bone in a fraction of the time, it does not require the sample to be decalcified, and it is a non-destructive technique that leaves the sample intact for further processing and examination^13,14^. Micro-CT is a relatively expensive technique which, in combination with the costs associated with using large numbers of animals for research studies, adds to the difficulty in generating large datasets. Therefore, in recent years, increasing efforts have been made to make datasets, produced through a variety of techniques, available to the public to allow further analysis of previously acquired data or to allow comparison of this data to new results. Moreover, micro-CT parameters have been standardised and guidelines on image acquisition, processing and analysis are available^14,15^ and widely adopted, therefore making data sharing and comparison easier for researchers. Scans and 3D reconstructions can be shared via their deposition in online repositories which assign a DOI for referencing to each file. Researchers are making increasing efforts to deposit their micro-CT data online for public access and various groups in the field of skeletal research have already created databases of micro-CT reconstructions, including non-human primate skulls and teeth^16,17^.

We have made the micro-CT dataset used for the analysis of bone microarchitecture of WT, *oim* mice and *oim* mice transplanted with human amniotic fluid mesenchymal stem cells (hAFSCs) available by Figshare, an open access repository. These methods are expanded versions of descriptions in our related work (Ranzoni *et al.*^18^). All experimental protocols complied with UK Home Office guidelines (PPL 70/6857). Briefly, heterozygous male and female mice (B6C3Fe a/a-Col1a2^oim^/Col1a2^oim^, Jackson Laboratory) were housed at 21° C with a 12:12-hour light/dark cycle. All mice were handled throughout the study by the same operator. It was the first pregnancy for all females. Pregnant females were housed individually. Offsprings were genotyped by sequencing the *oim* fragment and housed at 21° C with a 12:12-hour light/dark cycle with their mother. All mice used were experimentally naïve prior to this study. At 3-4 days of age, homozygous *oim* neonates from the same litter were randomly assigned to the transplanted or non-transplanted group and we ensured that mice from at least six different litters were included in all groups (wild-type, oim non-transplanted and oim transplanted). Homozygous oim neonates were individually intraperitoneally injected (between 10:00 and 12:00) with hAFSCs (10^6^ cells/mouse resuspended in 20μl cold PBS) under direct vision using a 33-G Hamilton Microlitre syringe (Bonaduz, Switzerland) without anaesthesia and replaced in their home cage with their mother immediately after injection. No blinding was implemented with regards to genotype. At the age of 30 days, mice were individually housed in filter cages (sawdust bedding) with a 12:12-hour light-dark cycle (21 °C), with water and chow (Purina, St Louis, MO) ad libitum. All experimental mice were culled at 8 weeks of age for analysis. The data set includes a total of 21 micro-CT mouse tibia axial scan sets including 6 wild-type mice, 6 *oim* and 9 *oim* transplanted with hAFSCs. Details on age and sex of the mice can be found in Table 1. One weakness of the study is that the oim non-transplanted group only includes males, whilst the two other groups (oim transplanted with stem cells and wild-type) contained both males and females; sex affects skeletal size and body weight. However, we have previously compared the effect of sex on the data reported by microCt, in particular bone mineral density, trabecular architecture and organisation, and found that sex had no significant impact on the microstructural properties assessed in the study. We have made this dataset accessible for re-use to the scientific community to encourage researchers to compare their datasets to ours and to therefore facilitate the investigation of new hypotheses.

## Methods

Tibiae were isolated from immune competent 8-week old wild-type mice (B6C3Fe a/a-Col1a2^+/+^) (n=6), homozygous *oim* mice (B6C3Fe a/a-Col1a2^oim/oim^) (n=6) and homozygous *oim* mice transplanted with hAFSCs (n=9) and fixed in 10% neutral buffered formalin for 24 h. Samples were then washed in phosphate buffered saline and stored in 70% ethanol until scanning.

**Figure 1:**
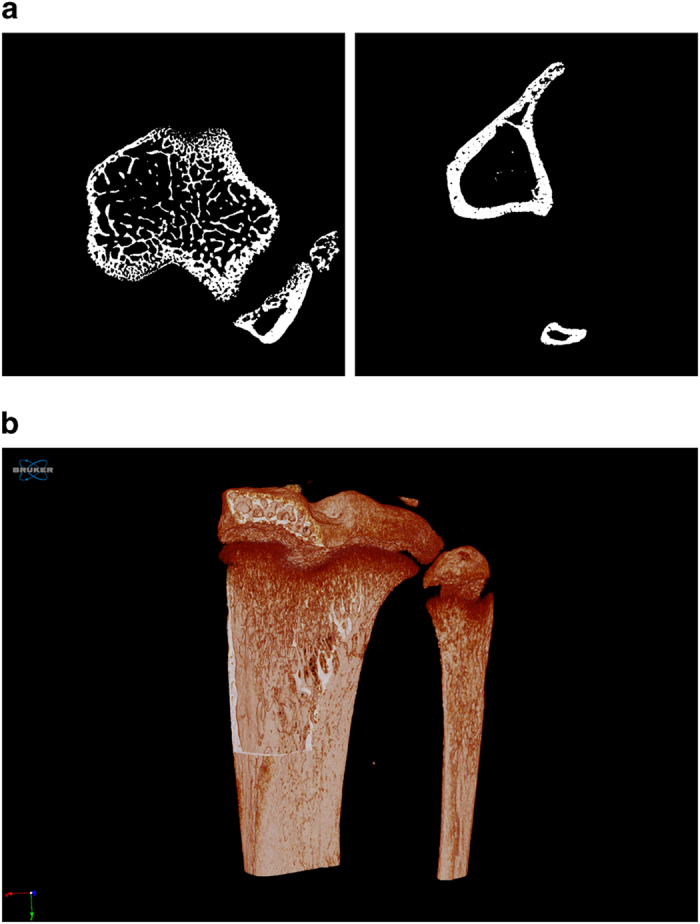
Example of reconstructed images obtained from the scans. Binarised cross-sectional images of trabecular and cortical bone of the tibia (**a**) and 3D rendering of the bone (**b**).

The bones were scanned with a Skyscan 1172 micro-CT scanner (Bruker, Coventry, UK), in small plastic tubes containing 70% ethanol. Double blinding was implemented during scanning measurement. The bones of wild type, oim non-transplanted and oim transplanted bones were randomly scanned to minimise any potential order effect. Scans were performed using a beam energy of 49KV and a flux 200μA, a 0.5mm aluminum filter and an isotropic pixel size of 5.06μm. The region scanned extended for 5 mm, starting from the proximal tibial epiphysis. We have compared the images and microstructure of oim bones scanned with frame averaging of 3 and frame averaging of 16 and found that the data related to microstructure were not significantly improved (data not shown). As there was no improvement in image quality, we have scanned all the bones of the study with a frame averaging of 3. Consequently, each image was acquired with an exposure time of 590ms, frame averaging of 3 and a 0.4 degree rotation step with a total rotation of 180 degrees and using medium camera resolution (2000x1048px). Random movement was set to ON and flat field correction was applied. Each scan includes a dataset of 991 stacked cross-sectional images, created for each scanned sample using NRecon segmentation software (Bruker) and stored as 8 bit Bitmap. The following settings were used for the reconstruction in NRecon: ring artifacts reduction was enabled and set to 1, smoothing was enabled and set to 1 to reduce background noise and beam hardening correction was enabled and set to 25%. Moreover, 3D reconstructions are available in the PLY format. Image stacks can be processed via CTAn software and 3D segmentation softwares CTVol and CTVox (Bruker) and via BoneJ, an online tool which can be downloaded for free^19^. Examples of reconstructed BMP images and 3D renderings obtained from CTVox are shown in Fig. 1.

## Data Records

The microCT dataset (Data Citation 1) comprises 21 sets of micro-CT scans of the proximal part of the tibia from 21 mice, including 6 from wild-type mice, 6 from non-transplanted *oim* mice and 9 hAFSCs-transplanted *oim* mice. Each scan set contains 991 stacked cross-sectional BMP images (approximate size of each folder 900MB). Additionally, 3D reconstructions are provided in the PLY format (Polygonal File Format).

## Technical Validation

The micro-CT scanner Skyscan 1172 is regularly serviced by Bruker UK engineers to verify the stability of the x-ray source. Density calibration was performed applying the Hounsfield unit method, before density measurement.

## Usage Notes

Researchers are free to download the files directly from Figshare (Data Citation 1), and to collect their own data from them.

## Additional information

**How to cite this article:** Ranzoni, A. M. *et al.* Micro-computed tomography reconstructions of tibiae of stem cell transplanted osteogenesis imperfecta mice. *Sci. Data* 5:180100 doi: 10.1038/sdata.2018.100 (2018).

**Publisher’s note:** Springer Nature remains neutral with regard to jurisdictional claims in published maps and institutional affiliations.

## Supplementary Material



## Figures and Tables

**Table 1:** Characteristics of animals used in the study.

**Animal**	**Sex**	**Age**	**Weight**
Wild-type 1	F	59 Days	18.3 g
Wild-type 2	M	59 Days	23.2 g
Wild-type 3	M	58 Days	23.5 g
Wild-type 4	M	58 Days	23.0 g
Wild-type 5	F	58 Days	18.5 g
Wild-type 6	F	58 Days	18.1 g
Oim 1	M	58 Days	17.3 g
Oim 2	M	58 Days	17.5 g
Oim 3	M	59 Days	18.0 g
Oim 4	M	59 Days	16.5 g
Oim 5	M	58 Days	16.5 g
Oim 6	M	58 Days	17.1 g
Transplanted 1	M	58 Days	17.1 g
Transplanted 2	M	58 Days	17.8 g
Transplanted 3	F	59 Days	15.1 g
Transplanted 4	M	58 Days	17.7 g
Transplanted 5	M	58 Days	17.5 g
Transplanted 6	F	58 Days	14.6 g
Transplanted 7	M	58 Days	16.7 g
Transplanted 8	M	58 Days	17.0 g
Transplanted 9	F	58 Days	15.4 g
The table shows the details of the wild-type, *oim* and transplanted *oim* mice, including sex, age and weight.			
